# Salivary histatin 5 internalization by translocation, but not endocytosis, is required for fungicidal activity in *Candida albicans*

**DOI:** 10.1111/j.1365-2958.2010.07210.x

**Published:** 2010-06-11

**Authors:** Woong Sik Jang, Jashanjot Singh Bajwa, Jianing N Sun, Mira Edgerton

**Affiliations:** Department of Oral Biology, School of Dental Medicine, State University of New York at BuffaloBuffalo, NY 14214, USA

## Abstract

Salivary histatin 5 (Hst 5) is a cationic salivary protein with high fungicidal activity against *Candida albicans*. Binding to the cell wall followed by intracellular translocation is required for killing; however, specific binding components and critical toxic events are not understood. In this study, laminarin (β-1,3-glucan) but not sialic acid, mannan or pustulan mediated Hst 5 binding to *C. albicans*, and was disassociated by 100 mM NaCl. Time-lapse confocal microscopy revealed a dose-dependent rate of cytosolic uptake of Hst 5 that invariably preceded propidium iodide (PI) entry, demonstrating that translocation itself does not disrupt membrane integrity. Cell toxicity was manifest by vacuolar expansion followed by PI entrance; however, loss of endocytotic vacuolar trafficking of Hst 5 did not reduce killing. Extracellular NaCl (100 mM), but not sorbitol, prevented vacuolar expansion and PI entry in cells already containing cytosolic Hst 5, thus showing a critical role for ionic balance in Hst 5 toxicity. Hst 5 uptake, but not cell wall binding, was blocked by pretreatment with azide or carbonyl cyanide m-chlorophenylhydrazone; however, 10% of de-energized cells had membrane disruption. Thus, Hst 5 is capable of heterogeneous intracellular entry routes, but only direct cytosolic translocation causes cell death as a result of ionic efflux.

## Introduction

Innate immunity is the first line of host defence against invasion by a wide spectra of bacteria and fungi (Hoffmann *et al*., 1999). Several proteins with antimicrobial activities are produced in the human oral cavity and contribute to innate host defences (Baev *et al*., 2002). Of these, the most potent antifungal member is salivary Histatin 5 (Hst 5). Hsts are a family of small histidine-rich cationic peptides secreted from human parotid and submandibular salivary glands (Oppenheim *et al*., 1988). Within this group, Hst 5 (24 amino acids) has the highest killing activity against *Candida albicans* at physiological concentrations (15–30 µM), and is also fungicidal to other pathogenic *Candida* species (Raj *et al*., 1990; Castagnola *et al*., 2004). Hsts have become the focus of considerable interest as candidate therapeutic agents for oral candidiasis, a common infection by the opportunistic yeast pathogen *C. albicans*, because of their high candidacidal and candidastatic activities while being nontoxic to humans (Edgerton *et al*., 1998; Jang *et al*., 2008).

The mechanism by which Hst 5 causes fungal cell death has been the subject of considerable controversy. Although there is a general agreement that Hst 5 must initially interact with and pass through the fungal cell wall before reaching its target to induce toxicity, the mode of interaction with the cell wall and membrane as well as the ultimate target within yeast cells have been disputed. Early work identified *C. albicans* mitochondria as the target of Hst 5 based upon its colocalization with these organelles. The observed reduction in susceptibility to Hst 5 by mitochondrial (‘petite’) mutants and cells treated with energy inhibitors such as azide and carbonyl cyanide m-chlorophenylhydrazone (CCCP) (Gyurko *et al*., 2000) also suggested the requirement of intact mitochondria for Hst 5 activity. The mechanism of cell death was postulated not to be a result of loss of respiration, because *C. albicans* are fully functional under fermentative (anaerobic) conditions, but rather to be a direct result of generation of toxic levels of reactive oxygen species from disrupted mitochondria (Helmerhorst *et al*., 2001). However, others found no role for ROS (reactive oxygen species) in Hst 5-induced yeast cell death (Veerman *et al*., 2004), and subjecting cells to oxidative stress did not enhance Hst 5 killing (Vylkova *et al*., 2007) suggesting that oxidative stress responses are secondary events in Hst 5 toxicity.

Other features of Hst 5 toxicity are cell permeability to ions and nucleotides. Early markers of fungicidal effects of Hst 5 on *Candida* cells are non-cytolytic efflux of cellular ATP (Koshlukova *et al*., 1999), potassium and magnesium ions (Xu *et al*., 1999), but not the passage of larger anionic dyes such as calcein (Edgerton *et al*., 1998; Helmerhorst *et al*., 1999). Our work identified alteration of cell volume as an early marker of Hst 5 toxicity, implicating disruption of cellular ionic balance but not membrane integrity, as a crucial event in Hst 5 fungicidal activity (Baev *et al*., 2002). We also identified *C. albicans* Trk1 membrane potassium transporters as an anion pathway for Hst 5-induced ATP loss (Baev *et al*., 2004). Although evidence for direct disruption of Trk1 function by Hst 5 is lacking, Trk1p is required for Hst 5-mediated loss of intracellular ions and nucleotides. Furthermore, *C. albicans hog1* mutants that lack osmotic stress responses are hypersensitive to Hst 5 (Vylkova *et al*., 2007), thus showing a central role of Hst 5-induced osmotic stress for cytotoxicity.

Hst 5 initially binds to selective components with the fungal cell wall, then must be translocated into the cytosol to have toxic effects (Xu *et al*., 1999; Koshlukova *et al*., 2000; Baev *et al*., 2002; Mochon and Liu, 2008). Previously, we found that digestion of the *Candida* cell wall to produce spheroplasts reduced killing by Hst 5 (Edgerton *et al*., 1998), and incubation of cells in high ionic-strength buffers also reduced levels of cell-associated Hst 5 (Jang *et al*., 2008). We identified *C. albicans* Ssa2p cell wall proteins (Li *et al*., 2006; Sun *et al*., 2008) to be involved in Hst 5 binding, although mutants deleted of these proteins retained partial binding activity. Thus it is likely that binding to the cell surface is also mediated by cell wall components, perhaps carbohydrates (Jang *et al*., 2006).

Approximately, 80–90% of the *C. albicans* cell wall is carbohydrate that protects cells from osmotic stress and maintains structural integrity (Martinez *et al*., 1998). The outermost layer is enriched in polymers of mannose (mannan) and covalently bound mannoproteins (Kapteyn *et al*., 2000). Underlying this layer are branched polymers of glucose containing β-1,3 and β-1,6 linkages (β-glucans), while the most inner layer near the membrane is composed of unbranched polymers of N-acetyl-D-glucosamine (GlcNAc) containing β-1,4 bonds (chitin) (Chaffin *et al*., 1998). Initial binding of Hst 5 to the *C. albicans* cell wall is disrupted by elevation of extracellular salt concentrations above 100 mM (Helmerhorst *et al*., 1999; Xu *et al*., 1999; Jang *et al*., 2008), thus it has been difficult to differentiate loss of binding from downstream effects of Hst 5 that involve intracellular ionic imbalance. Therefore in this study, we designed experiments that separate the effects of disruption of Hst 5 binding and translocation by extracellular salt from subsequent effects involving ionic stabilization to understand the role of ion loss for Hst 5 killing.

Endocytotic transport of Hst 5 is a potential route for intracellular Hst 5 uptake and for its fungicidal activity in *C. albicans*. In fungi, rapid changes in vacuole size are a result of uptake or release of water and solutes in order to balance cytoplasmic ion concentrations upon osmotic stress (Efe *et al*., 2005). Under hypo-osmotic conditions the cell vacuole enlarges, while hyper-osmotic conditions induce vacuolar fragment and shrinkage (Hohmann, 2002; Efe *et al*., 2005); thus the expansion of the central vacuole found following Hst 5 treatment (Mochon and Liu, 2008) may reflect changes in ionic equilibrium as a result of loss of cytosolic ions. In addition, the vacuole is the site of storage of selected amino acids and phosphate, metals and some toxins (Weisman, 2003). Interestingly, energy-dependent endocytosis was found to remove Hst 5 from the cell surface to the vacuole and reduce killing (Mochon and Liu, 2008), suggesting a further role of the cell vacuole in detoxification. Thus, endocytotic transport of Hst 5 to the yeast vacuole may have a role in re-establishing ion homeostasis in addition to sequestering Hst 5, both of which may reduce Hst 5 toxicity. However, the total contribution of endocytosis to Hst 5 killing in *C. albicans* is not known.

Recent attention has been focused on the role of the cell membrane and energetics in Hst 5 uptake and killing. Sodium azide pretreatment of *C. albicans* caused cells to become resistant to the killing action of Hst 5 as a result of energy depletion and loss of intracellular ATP (Koshlukova *et al*., 1999; Veerman *et al*., 2007). Pretreatment with the protonophore CCCP that collapses the yeast plasma membrane proton electrochemical gradient also protected *C. albicans* from Hst 5 (Koshlukova *et al*., 1999; Gyurko *et al*., 2000), perhaps because the plasma membrane proton gradient is necessary for action of phospholipid flippases (Stevens and Nichols, 2007) that regulate endocytosis. Others found that azide blocked both Hst 5 interaction with the *C. albicans* cell wall and its internalization, but attributed this effect to cell membrane rigidification due to ATP depletion (Veerman *et al*., 2007). In contrast, Mochon and Liu (Mochon and Liu, 2008) reported that Hst 5 acts on the plasma membrane directly to form a single breach site allowing Hst 5 entry. This group found that membrane breach required an electrochemical gradient and was the primary event in killing (Mochon and Liu, 2008). However, others have reported no disruption of phospholipid membranes by Hst 5 and no translocation across liposome membranes (Den Hertog *et al*., 2004), rendering the mechanistic basis for a single membrane pore unclear.

The objectives of the present study were to determine the relative contribution of translocation and endocytosis for Hst 5 toxicity in *C. albicans* and to delineate the role of the cell vacuole and osmotic balance for Hst 5 toxicity. Time-lapse confocal microscopy was used to understand the dynamic process of Hst 5 trafficking through the cell wall and membrane, and showed that translocation but not endocytosis is required for Hst 5 toxicity. We identified salt-sensitive binding of Hst 5 to cell wall β-glucans and cell energy as crucial for Hst 5 intracellular translocation. In addition, we report unequivocal evidence that intracellular translocation of Hst 5 does not occur simultaneously with PI uptake or pore formation, rather PI uptake is a consequence of ionic efflux and vacuole expansion induced by cytosolic Hst 5, implicating ionic imbalance as the central event leading to cell death.

## Results

### Hst 5 binding to β-glucans on the cell wall of *C. albicans* is salt-sensitive

**Fig. 1:**
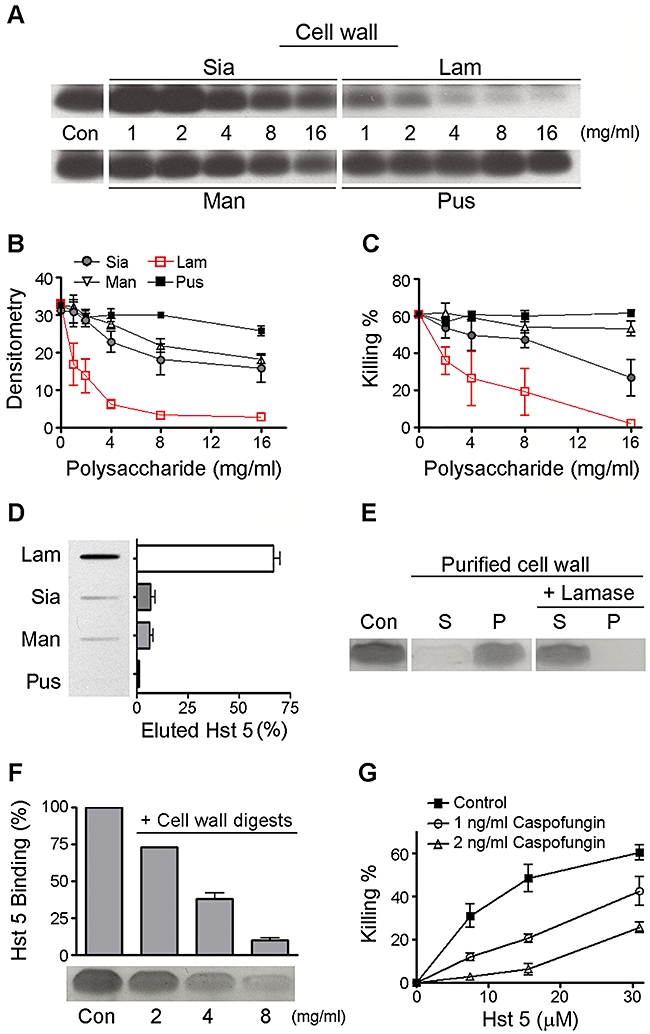
Hst 5 binds selectively to β-1,3-glucans in the *Candida* cell wall. Representative cell wall polysaccharides (1–16 mg ml^−1^) consisting of laminarin (β-1,3-glucan) (Lam, red squares), sialic acid (Sia, grey circles), mannans (Man, black diamonds), pustulan (β-1,6-glucan) (Pus, black squares) or untreated control (Con) were pre-incubated with BHst 5 (31 µM) then added to *C. albicans* cells for 1 h. Cell wall extracts were immunoblotted to detect BHst 5 (A) and quantified by dentistometry (B). Reduction in cell wall binding was accompanied by reduced candidacidal activity of Hst 5 when incubated with the same polysaccharides in a dose-dependent manner (C). The same polysaccharides were coupled to Sepharose beads and percent binding of BHst 5 was assessed by elution affinity chromatography (D). Only laminarin was found to have significant binding with Hst 5. PCW and laminarinase-treated PCW were incubated with Hst 5, and unbound Hst 5 in supernatant (S) and bound Hst 5 in pellets (P) were detected on Tricine-SDS gels (E). Laminarinase digestion of PCW prevented Hst 5 binding (E). Cell wall binding of Hst 5 was reduced in a dose-dependent manner by laminarinase digests (F), when laminarinase digests (2–8 mg ml^−1^) were pre-incubated with Hst 5, then added to *C. albicans* cells. Killing activities of Hst 5 were decreased against *C. albicans* cells grown in YPD broth with caspofungin (1 or 2 ng ml^−1^) (G).

To determine whether Hst 5 binding has any specificity with cell wall polysaccharides, we assessed biotin-labelled Hst 5 (BHst 5) cell wall binding and killing activity in the presence of carbohydrates representative of major fungal cell wall polysaccharides (Fig. 1A, B and C). BHst 5 (31 µM) was pre-incubated with laminarin (β-1,3-glucan, MW = 6 kDa), sialic acid, mannan or pustulan (β-1,6-glucan) (1–16 mg ml^−1^) prior to addition to *Candida* cells. Pre-incubation of BHst 5 with laminarin (4 mg ml^−1^) decreased cell wall binding of BHst 5 by approximately 80%; while pre-incubation of BHst 5 with mannan, sialic acid or pustulan had no effect on cell wall binding (Fig. 1A and B). Addition of higher concentrations of sialic acid or mannan (16 mg ml^−1^) reduced BHst 5 binding with the cell wall by 48.5% and 55.7% respectively; while pustulan still had no effect on cell wall binding of BHst 5. As expected, Hst 5 killing was reduced in proportion with its loss of cell wall binding as the amount of laminarin was increased in the mixture (Fig. 1C, red line). Killing activity of Hst 5 was reduced by half to one quarter in the presence of sialic acid and mannan (16 mg ml^−1^), but pustulan did not affect its fungicidal activity.

To determine the relative specificity of binding between Hst 5 and laminarin, carbohydrate-conjugated Sepharose columns were used to bind Hst 5 then measure its elution with salt. BHst 5 (100 µg) was applied to laminarin-, sialic acid-, mannan- or pustulan- conjugated Sepharose 6B columns, washed, then bound BHst 5 was eluted with 10 mM, sodium-phosphate buffer (pH 7.4), containing 100 mM NaCl as this represents physiogical conditions (Fig. 1D). Bound BHst 5 recovered from the laminarin column with 100 mM NaCl was 66.8% of the applied peptide, and all peptide was removed with 1 M NaCl. In contrast, less than 10% of applied BHst 5 was bound to sialic acid, mannan or pustulan columns. Thus, Hst 5 binds strongly and selectively with laminarin, and physiological levels of salt can disassociate binding.

*Candida albicans* cell walls were isolated and purified for use in Hst 5-binding assays. When purified cell walls were added to Hst 5, nearly all Hst 5 was recovered with cell wall pellets following centrifugation. However, laminarinase digestion of purified cell walls resulted in complete loss of Hst 5 binding (Fig. 1E). Next, purified cell walls were subjected to laminarinase digestion and released polysaccharides were recovered. Column chromatography of carbohydrates released by laminarinase digestion showed a single peak of 7 kDa consistent with a 40 unit glucose polysaccharide. This digested cell wall β-glucan (2–8 mg ml^−1^) was added to the incubation mixture to compete with Hst 5 binding to intact *C. albicans* cells. Laminarinase digests were able to compete for binding to cells with Hst 5 in a dose-dependent manner, so that nearly complete inhibition of Hst 5 binding was observed in the presence of 8 mg ml^−1^ of laminarinase digests (Fig. 1F). In contrast, addition of laminarin biose and triose did not compete with Hst 5 binding (data not shown), demonstrating that Hst 5 binding to cell walls involves more complex β-glucans rather than di- or tri-saccharides.

Caspofungin is an antifungal drug that specifically inhibits β-(1,3)-D-glucan synthase, and results in specific cell wall defects in *C. albicans* as a result of loss of β-1,3-glucans. To determine if reduction in cell wall β-1,3-glucans altered susceptibility to Hst 5, cells were grown overnight in the presence of sub-inhibitory doses of caspofungin. These cells grown in the presence of 1 or 2 ng ml^−1^ of caspofungin had a dose-dependent reduction in killing by Hst 5 (Fig. 1G) as result of reduced Hst 5 cell wall binding, indicating that β-1,3-glucans are binding sites for Hst 5 *in vivo*.

### Kinetics of uptake of Hst 5 show dose-dependent translocation and intracellular accumulation precede vacuole expansion and PI uptake

**Fig. 2:**
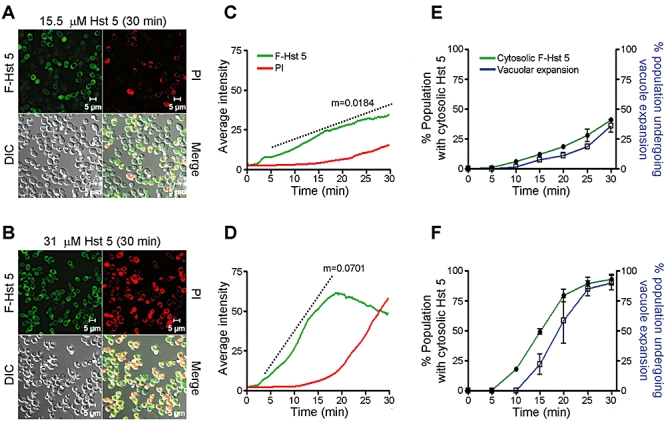
Uptake rate of Hst 5 into *C. albicans* cells is time- and concentration-dependent. The average cytosolic concentration of F-Hst 5 [15.5 µM (A); or 31 µM (B)] and the average cytosolic intensity of PI were measured in a cell population (≈ 200 cells) by time-lapse confocal microscopy. F-Hst 5 was added to *C. albicans* cells in 10 mM NaPB buffer containing PI (5 µg ml^−1^) and images were recorded every 10 s for 30 min. Total cytosolic F-Hst 5 (green) increased linearly (slope = m) over 30 min when applied at 15.5 µM (C) while little PI (red) uptake was evident until 25 min after Hst 5 addition. In contrast, the rate of uptake (m) of F-Hst 5 more than doubled by increasing the peptide concentration to 31 µM (D) up to 20 min; after which total cytosolic Hst 5 began to decrease. No PI uptake (red) occurred until substantial cytosolic levels of Hst 5 were reached (20 min), and PI entry was coincident with decreasing levels of cytosolic Hst 5. Number of cells containing cytoplasmic F-Hst 5 and the number of cells having expanded vacuoles were recorded as a percentage of the total cell population every 5 min following addition of 15.5 µM F-Hst 5 (E) or 31 µM F-Hst 5 (F). The percentage of cells with vacuolar expansion (blue) was the same as the percentage of cells with cytosolic F-Hst 5 (green) for both concentrations of Hst 5 (E, F), except that there was a 5 min lag for cells undergoing vacuolar expansion.

Although we previously found that intracellular localization is a requirement for the toxic effects of Hst 5 (Li *et al*., 2006; Jang *et al*., 2008), very little is known about early Hst 5 trafficking or levels of intracellular accumulation necessary for killing. To examine the temporal relationship of trafficking of Hst 5 into cytoplasm with the initiation of PI influx, intracellular translocation of two physiological concentrations of F-Hst 5 (15.5 µM and 31 µM) were recorded using time-lapse confocal microscopy in a population of 200–250 *C. albicans* cells (Fig. 2A and B, Videos S1 and S2). F-Hst 5 was associated with cells within 2–3 min (Videos S1 and S2), and its time-dependent average intensity within the cytosol increased in proportion with applied F-Hst 5 concentrations. The average uptake rate (shown for 200 *C. albicans* cells) of 15.5 µM doses of F-Hst 5 was linear (slope = 0.0184) over 30 min, while the average uptake rate of 31 µM doses of F-Hst 5 was higher (slope = 0.0701) and linear only for the initial 15 min, after which the average uptake rate reached a plateau (Fig. 2C and D). Thus, 31 µM of applied F-Hst 5 resulted in both more rapid uptake of peptide as well as saturation of cells. Strikingly, no PI uptake was detected in cells at either F-Hst 5 concentration until 20–25 min following addition of peptide (Fig. 2C and D, Videos S1 and S2). In addition, intracellular PI accumulation was not observed until uptake rates reached plateau levels of F-Hst 5 (31 µM) (Fig. 2D). These data clearly show that PI entry occurs well after significant intracellular accumulation of Hst 5, and rules out membrane perturbation associated with Hst 5 entry into cells. Thus, PI entry is secondary to rather than concurrent with Hst 5 uptake.

It was also observed that many cells exhibited significant vacuolar expansion beginning 15–20 min post-treatment of both 15.5 µM and 31 µM F-Hst 5. To determine the kinetics of this reaction, the number of cells within the total population undergoing vacuolar expansion was quantitated and compared with the percentage of cells containing F-Hst 5 (Fig. 2E and F show averages of three experiments each using 200–250 cells). As expected for dose-dependent translocation, 45% of cells contained cytosolic Hst 5 (15.5 µM F-Hst 5) and 95% of cells contained F-Hst 5 at 31 µM doses after 30 min incubation. However, over the first 25 min after addition of Hst 5, the population of cells showing vacuolar expansion was delayed by 5 min at either F-Hst 5 concentration, showing that vacuolar expansion occurs after cytosolic accumulation of Hst 5. To confirm and further understand this relationship, we analysed early intracellular trafficking events of Hst 5 within single cells.

### Entry of Hst 5 into *C. albicans* occurs by both translocation and endocytosis

**Fig. 3:**
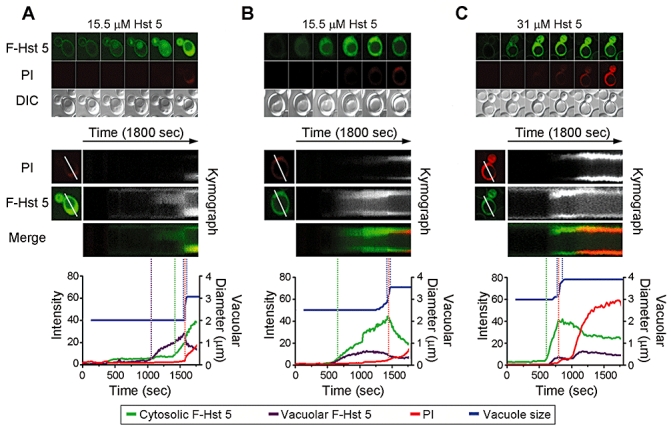
Hst 5 trafficking to the cytosol or vacuole precedes vacuolar expansion and PI uptake. Intracellular trafficking of 15.5 µM (A and B) and 31 µM (C) F-Hst was measured in parallel with PI uptake in single live cells by time-lapse confocal microscopy. Upper panels are recorded at 300, 600, 900, 1200, 1500 and 1800 s and illustrate two distinct trafficking routes of Hst 5 into cells, either vacuolar to cytosolic (shown in A with 15.5 µM F-Hst 5) or direct cytosolic (shown in B and C). Middle panels are kymographs composed of two-colour merged images of single-channel recordings (F-Hst 5 = green and PI = red) of each cell (1 frame/10 s). Fluorescence intensities of cytosolic F-Hst 5 (green), vacuolar F-Hst 5 (brown) and PI (red) are plotted with vacuolar size (blue) below each corresponding kymograph. Dotted lines indicate initial times of uptake of F-Hst 5 and PI and time of initiation of vacuolar expansion. For initial vacuolar trafficking at lower doses of Hst 5 (left panels, A), accumulation in the vacuolar occurred at 1000 s, followed by diffusion of the peptide to the cytosol (kymograph); however, independent cytosolic trafficking of Hst 5 also occurred. Vacuolar expansion and then PI uptake were initiated at 1600 s. Cytosolic trafficking of Hst 5 (B, 15.5 µM) in which the majority of peptide localized to the cytoplasm occurred at 600 s before initiation of vacuolar expansion and PI entry. Higher doses of Hst 5 (C, 31 µM) followed the same cytosolic trafficking pathway, but occurred more rapidly. In all cases, vacuolar expansion occurred only after significant cytosolic levels of intracellular Hst 5 were reached, followed by PI uptake.

Intracellular trafficking of F-Hst 5 (15.5 µM and 31 µM) was analysed in real time in parallel with vacuolar diameter and PI uptake in individual cells (Fig. 3). We observed two distinct pathways of Hst 5 uptake among a population of Hst 5-treated cells, and selected representative single cells (from a total population of 200–250 cells) for analysis of the kinetics of Hst 5 uptake. In one pathway (Fig. 3A), F-Hst 5 was transported to the cell vacuole by endocytosis. This vacuolar pathway was observed in approximately 70% of cells treated with 15.5 µM Hst 5 and 40% of cells exposed to 31 µM Hst 5 (see Videos S1 and S2). Compartmental levels of F-Hst 5 (shown in kymographs and quantitated below) revealed that vacuolar accumulation of F-Hst 5 (15.5 µM) was relatively slow, beginning at 1000 s following addition of Hst 5. Vacuolar expansion occurred at approximately 1500 s after addition of F-Hst 5 (blue line), and was accompanied by release of vacuolar F-Hst 5 to the cytosol (green line) and initiation of PI entry (red line). Strikingly, nearly complete loss of vacuolar Hst 5 into the cytoplasm (Fig. 3A, kymograph) occurred simultaneously with vacuolar expansion, suggesting that the vacuolar membrane becomes more permeable to small molecules such as Hst 5.

In a second entry pathway, F-Hst 5 was directly translocated into the cytoplasm (Fig. 3B and C) with virtually no vacuolar accumulation. Direct cytoplasmic translocation of Hst 5 occurred more rapidly, so that cytosolic Hst 5 was detected at 500 s following addition of either concentration of Hst 5. However, as observed for whole cell populations (Fig. 2), single-cell levels of cytoplasmic Hst 5 increased more rapidly at higher doses of Hst 5 (Fig. 3B and C, green lines). At 15.5 µM of applied F-Hst 5, vacuolar expansion also occurred at approximately 1500 s (Fig. 3B), while 31 µM F-Hst 5 induced expansion more rapidly at 750 s (Fig. 3C). In either case, PI uptake did not occur until vacuolar expansion was initiated, and in some instances well over 200 s afterwards, indicating that cell membrane integrity becomes compromised related to cellular events associated with an increase in vacuole size. Another finding from these time-lapse images is that vacuolar expansion is a rapid event that in some instances resulted in vacuole rupture (Videos S1–2).

### Localization of Hst 5 to *C. albicans* vacuoles does not reduce Hst 5 toxicity

**Fig. 4:**
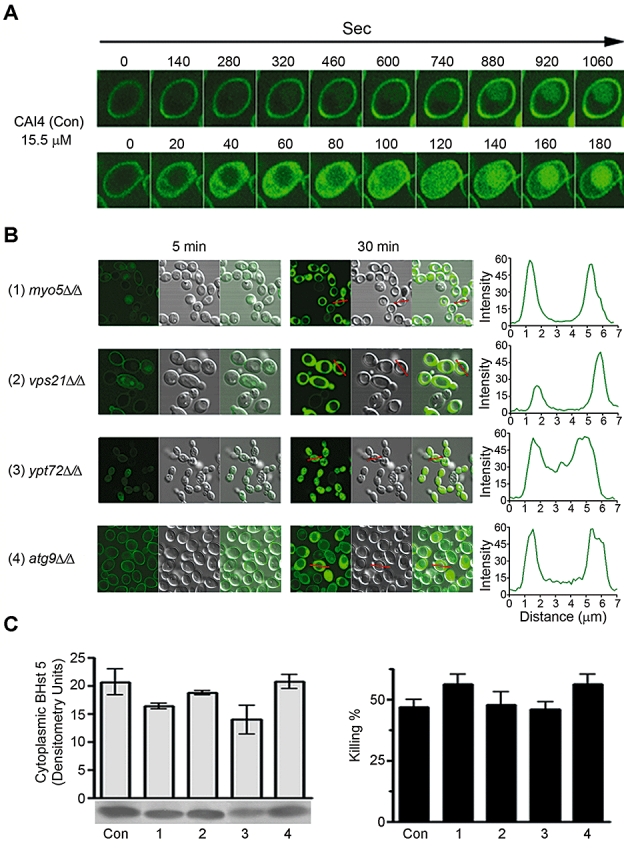
*C. albicans* cells with endocytotic and/or autophagy defects are capable of Hst 5 cytosolic uptake and are sensitive to Hst 5. Both slower endocytotic (A, upper panel) and more rapid autophagy (A, lower panel) trafficking of Hst 5 were observed in CAI4 control cells. Vacuolar trafficking of Hst 5 was examined in *C. albicans* endocytosis and autophagy mutants by time-lapse confocal microscopy at 5 min and 30 min after addition of 15.5 µM F-Hst 5 (B). Right panels show cross-sectional content of F-Hst 5 in individual cells (red lines). F-Hst 5 was trafficked to vacuoles in *myo5*Δ/Δ and *vps21*Δ/Δ endocytotic mutants within 5 min, and substantial cytosolic localization of F-Hst 5 was observed in *myo5*Δ/Δ and *vps21*Δ/Δ mutants as well as *ypt72*Δ/Δ (endocytosis defect) and *atg9*Δ/Δ (autophagy defect) mutants at 30 min. Western blot analyses (C, left panel) of cytosolic density of cells (10^7^) exposed to Hst 5 showed that all mutants had levels of cytosolic Hst 5 equal to CAI4 control cells (con: control). Similarly, all mutants were equally sensitive to Hst 5 killing as CAI4 control cells (C, right panel).

**Fig. 5:**
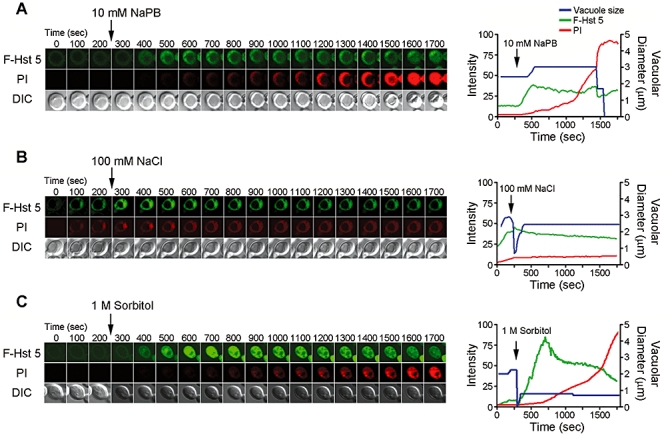
Addition of extracellular salt to *Candida* cells prevents cytosolic Hst 5 from inducing vacuolar expansion and PI uptake. *C. albicans* cells were incubated with 31 µM F-Hst 5 and 5 µg ml^−1^ PI for 5 min to allow cytosolic accumulation of peptide, then 10 mM NaPB (A), 100 mM NaCl (B) or 1 M sorbitol (C) was added to the cell suspension (indicated by arrows). Confocal image acquisition (1 frame per 10 s for 30 min) began immediately after the addition of F-Hst 5; and fluorescence intensities of cytosolic F-Hst 5 (green) and PI (red) and vacuole size (blue) were calculated (right panels). Control cells in 10 mM NaPB underwent vacuolar enlargement followed by PI influx. In contrast, cells treated with 100 mM NaCl (B) did not undergo vacuolar expansion and had no influx of PI despite having significant cytosolic concentrations of Hst 5. This effect was specific to extracellular ionic strength as addition of 1 M sorbitol (C) substantially increased F-Hst 5 intracellular translocation and allowed PI influx.

Since vacuolar effects of Hst 5 were observed, we hypothesized that cells with defects in endocytosis (cytoplasm to vacuole transport), cells with defects in autophagy transport to the vacuole, as well as cells carrying mutations resulting in the absence of vacuoles, all would be hypersensitive to Hst 5. Time-lapse confocal microscopy showed that Hst 5 utilized two distinct trafficking routes to reach the vacuole (Fig. 4A). Both vesicle-mediated endocytosis (Fig. 4A upper) and slower cytoplasmic to vacuole transport (autophagy) (Fig. 4A lower) were observed routes for F-Hst 5 (15.5 µM) trafficking to the vacuole. Therefore, we selected *C. albicans* mutants with defects of both endocytotic and autophagy pathways (Table 1) to examine for susceptibility to Hst 5. Endocytotic pathway mutants *myo5*Δ/Δ (Oberholzer *et al*., 2002)*, vps21*Δ/Δ (Johnston *et al*., 2009) both having normal vacuolar structures, *ypt72*Δ/Δ (Johnston *et al*., 2009) mutant lacking vacuoles and an autophagy mutant (*atg9*Δ/Δ) (Palmer *et al*., 2007) were tested for sensitivity to Hst 5. There were no differences in Hst 5 susceptibilities between CAI4 (control) and mutant strains based upon ura status. However, as mutations within endocytotic pathways may cause multiple cellular defects (Oberholzer *et al*., 2006), we verified cytosolic concentrations of Hst 5 by confocal microscopy (Fig. 5B right) and by Western blotting of total *C. albicans* cytosolic extracts (Fig. 4C left). Vacuolar localization of F-Hst 5 could be visualized within 5 min (Fig. 4B left) after peptide addition in three endocytosis mutants that retain vacuoles (*myo5*Δ/Δ, *vps21*Δ/Δ), showing that the autophagy pathway for Hst 5 trafficking to the vacuole remains intact despite defects in endocytosis. After 30 min incubation with F-Hst 5 (Fig. 4B middle), substantial cytosol internalization of peptide was found in both *myo5*Δ/Δ and *vps21*Δ/Δ cells. In addition, *ypt72*Δ/Δ cells lacking a central vacuole and the autophagy mutant *atg9*Δ/Δ that is defective in fusion of autophagosome vesicles with the vacuole, both exhibited substantial amounts of cytosolic F-Hst 5, showing that the presence of a vacuole alone is not crucial for peptide uptake. Cytoplasmic density of BHst 5 was measured by Western blotting in cells following 30 min exposure to peptide, and all mutants had statistically similar levels of cytosolic BHst 5 content (Fig. 4C left), showing that neither autophagy nor endocytosis pathways alone are required for Hst 5 intracellular translocation. Because one major function of vacuole is to degrade proteins, we expected that autophagy and endocytosis mutants would be more sensitive to Hst 5 than CAI4 control cells. However, no significant difference in sensitivity to Hst 5 was observed in any mutant cell that had control levels of cytosolic translocation (Fig. 4C right), showing that vacuolar sequestration or degradation of peptide is not a significant means of removal of toxic peptide from candidal cells. This was substantiated by experiments in which cells pre-incubated with endocytosis inhibiting drugs (Jasplakinolide, Latrunculin A and Cytochalasin A, 10 µM) had no significant alteration of Hst 5 uptake or killing (data not shown). Collectively, these data show that Hst 5 is trafficked to the vacuole by both endocytotic and autophagy pathways, but that vacuolar localization of Hst 5 does not modulate its toxicity. These results also suggested that vacuolar expansion observed following Hst 5 intracellular accumulation is secondary to its toxic effects. To directly test this question, we subjected cells to extracellular osmotic stabilization following initial entry and accumulation of Hst 5.

**Table 1:** Strains used in this study.

Strain	Relevant genotype	Endocytotic defect	Source
CAI4 (control)	*ura3*Δ::*imm434/ura3*Δ::*imm434*	None	(Fonzi and Irwin, 1993)
*myo5*Δ/Δ	*myo5*Δ::*hisG/myo5*Δ::*hisGura3*Δ::*imm434/ura3Δ*::*imm434*	cortical actin patch + endocytosis	(Oberholzer *et al*., 2002)
YJB6284 (wild type)	*VPS21*/*VPS21 YPT72*/*YPT72ATG9/ATG9 URA3/URA3*	None	(Bensen *et al.*, 2002)
*vps21*Δ/Δ	*vps21*Δ::*ARG4/vps21*Δ::*HIS1ura3*Δ/Δ*his1*Δ/Δ*arg4*Δ/Δ	lack PVC, defects in surface PVC	(Johnston *et al*., 2009)
*ypt72*Δ/Δ	*ypt72*Δ::*ARG4/ypt72*Δ::*HIS1ura3*Δ/Δ*his1*Δ/Δ*arg4*Δ/Δ	intact PVC, no vacuole, defects in fusion of endosomes to vacuole	(Johnston *et al*., 2009)
*atg9*Δ/Δ	*atg9*Δ::*ARG4/atg9*Δ::*HIS1ura3*Δ/Δ::*URA3 his1*Δ/Δ*arg4*Δ/Δ	defects in fusion of PAS with vacuole	(Palmer *et al*., 2007)

PVC: prevacuolar compartment, PAS: pre-autophagosomal structure.

### Toxicity of cytoplasmic Hst 5 is prevented by high extracellular salt but not high extracellular osmolarity

Because extracellular salt (100 mM) prevents binding of Hst 5 to the cell wall, an alternative approach was needed to distinguish the effects of extracellular salt on Hst 5 cell wall binding from its ability to provide osmotic/osmolar stabilization. For these purposes, we allowed F-Hst 5 uptake to occur for about 250 s in order to reach a significant cytosolic concentration typically associated with vacuolar expansion [≈ 40 intensity units (IU)] before addition of extracellular salt or sorbitol (Fig. 5). Control cells (10 mM NaPB added after cells were exposed to Hst 5) underwent typical cytosolic accumulation of peptide to ≈ 40 IU, followed by vacuolar enlargement and then PI influx. In contrast, PI uptake did not occur in cells treated with 100 mM NaCl that showed rapid loss, then recovery of vacuolar size, while cytosolic levels of F-Hst 5 remained stable (Fig. 5B). As expected, extracellular salt prevented further F-Hst 5 uptake (as shown by flat intracellular Hst 5 IU); however, the presence of 100 mM NaCl also prevented Hst 5-induced cell toxicity as measured by PI uptake. Thus, increasing ionic strength of the extracellular environment prevented killing by intracellular Hst 5, showing that the central event leading to cell death is related to loss of cellular ion balance.

**Fig. 6:**
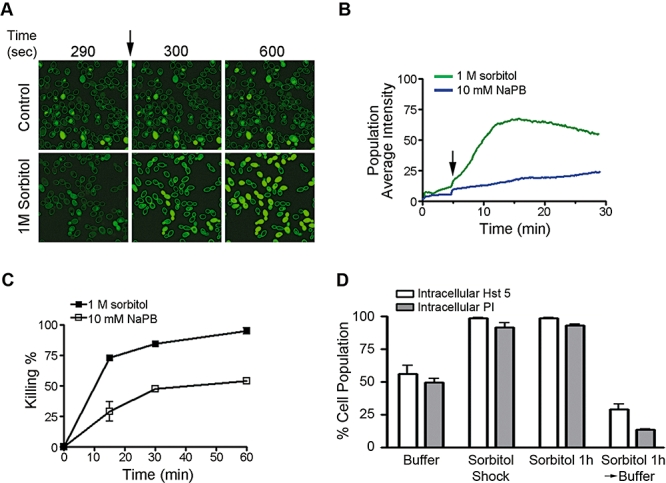
Osmotic shock and high extracellular osmolarity increase cytosolic uptake of Hst 5 and its fungicidal activity. Confocal images were acquired following addition of F-Hst 5 (15.5 µM) to 200 cells for 5 min, then 10 mM NaPB or 1 M sorbitol was added to the cell suspension (A, arrow); and total fluorescence intensity of F-Hst 5 was quantitated (B). Cells pretreated with F-Hst 5 followed by addition of 1 M sorbitol (green line) had more rapid accumulation of peripheral and intracellular Hst 5 when compared with 10 mM NaPB-treated cells (blue line). Candidacidal activity (C) of Hst 5 was increased in 1 M sorbitol-treated cells (closed squares) by more than twofold when compared with 10 mM NaPB-treated CAI4 control cells (open squares). The percentage of cells containing intracellular F-Hst 5 or PI after 30 min incubation with F-Hst 5 (15.5 µM) was quantified (D) in cells following sorbitol-shock (1 M sorbitol for 5 min), in cells pretreated with 1 M sorbitol for 1 h (sorbitol 1 h), or cells that were pretreated with 1 M sorbitol and than washed and suspended in with 10 mM NaPB (Sorbitol 1 h → Buffer). Cellular uptake of Hst 5 (white bars) and PI accumulation (grey bars) was increased substantially in cells either pretreated with 1 M sorbitol or in osmotically shocked cells. In contrast, cells conditioned with 1 M sorbitol then placed in 10 mM NaPB had nearly twofold less F-Hst 5 uptake and PI staining.

To determine whether protection from Hst 5 toxicity could be provided by extracellular osmolarity independently from ionic strength, 1 M sorbitol was added to cells following F-Hst 5 uptake (Fig. 5C). In a classical response to hyperosmotic stimuli, cell vacuoles contracted and remained smaller in size over the course of the experiment. However, in contrast to 100 mM NaCl, sorbitol substantially increased F-Hst 5 intracellular translocation even when added prior to cells reaching significant levels of F-Hst 5 (as shown in Fig. 5C). Sorbitol dramatically raised both the rate of F-Hst 5 translocation as well as total IU of cytosolic F-Hst 5 (up to 100 IU in many cells), although loss of intracellular F-Hst 5 was observed after 750 s in most cells. Interestingly, PI uptake was not initiated any earlier than for control cells despite nearly doubling of intracellular IU of F-Hst 5, implying a rate limiting step for interactions between some intracellular component with Hst 5. Thus, addition of sorbitol seemed to trigger an initial increase in intracellular Hst 5 transport rate. This was confirmed upon examination of cell populations pretreated with F-Hst 5 for 300 s (shown in Fig. 6A and B) followed by addition of 1 M sorbitol, that had rapid accumulation of peripheral and intracellular Hst 5 when compared with 10 mM NaPB-treated cells. The cumulative effect of increased Hst 5 uptake following 1 M sorbitol treatment of cells was reflected in nearly doubling Hst 5 toxicity (Fig. 6C). To determine whether this was due to hyperosmotic shock conditions alone, Hst 5 and PI uptake were evaluated in cell populations pretreated for 1 h with 1 M sorbitol compared with sorbitol-shocked cells pretreated for 5 min with 1 M sorbitol (Fig. 6D). Both treatments (pre-incubation with 1 M sorbitol and osmotic shock) resulted in similarly elevated cellular uptake of Hst 5 (98.5% and 98.7% respectively) and PI accumulation (92.9% and 91.5% respectively) compared with control (56.3% of cells contained Hst 5 and 49.4% of cells were stained with PI) suggesting that high osmolarity rather than osmotic shock was responsible for higher levels of intracellular Hst 5 and PI. In contrast, cells that had been pretreated for 1 h in 1 M sorbitol then washed with 10 mM NaPB buffer before addition of F-Hst 5 had normal cell binding but significantly reduced (*P* = 0.0359) F-Hst 5 uptake (28.8%) and PI staining (13.7%) compared with control, suggesting that cellular adaptation to hypotonic stress reduced Hst 5 translocation. In total, these results show that Hst 5 toxicity is prevented specifically by high ionic strength buffers and is not simply a result of osmotic protection. However, Hst 5 translocation itself is modulated by both high extracellular osmolarity (increased) and hypotonicity (decreased) independently from ionic strength. These results also point out that vacuolar expansion observed following Hst 5 treatment in low salt buffer is a consequence of altered cellular ionic balance (as vacuoles are highly responsive to ionic stress), but cells cannot recover from loss of ions and undergo cell death.

### Hst 5 can induce loss of cellular integrity with increased cell wall residency time

**Fig. 7:**
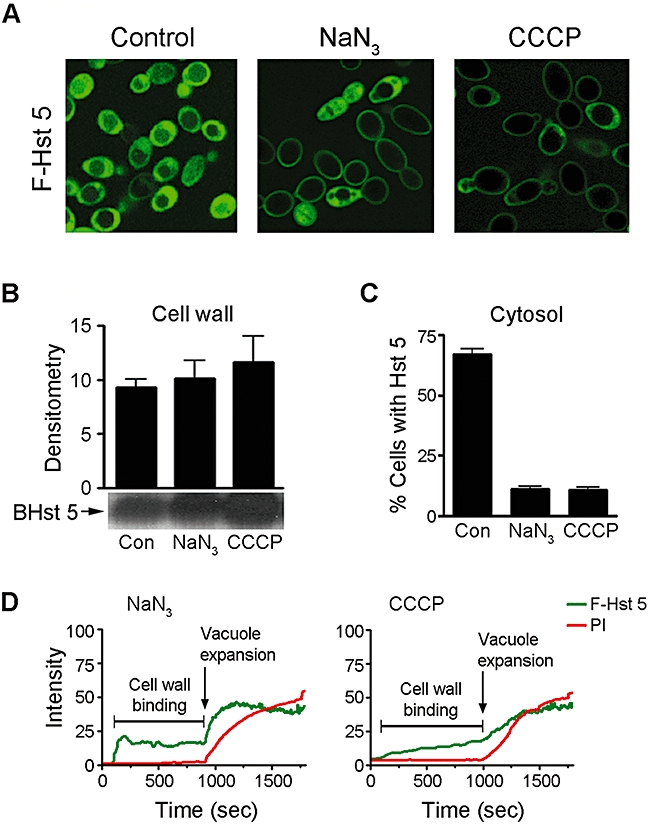
Energy depleted cells have prolonged cell wall retention of Hst 5. Azide (NaN_3_, 10 mM) and CCCP (500 µM) treated cells had equivalent amounts of cell wall bound BHst 5 as untreated controls (Con); but had reduced levels of cytosolic Hst 5 showing impaired Hst 5 uptake (A, B), although 11.2% and 10.9% of energy depleted *C. albicans* cells contained cytosolic Hst 5 respectively (C). Time-lapse confocal microscopy showed that F-Hst 5 was retained on the cell wall of both azide and CCCP pretreated cells; after which simultaneous entry of PI and Hst 5 occurred along with vacuolar expansion indicative of loss of membrane integrity (D).

It has been recognized that internalization of Hst 5 is energy-dependent, and both active transport and endocytotic processes require energy. Thus, either azide or CCCP treatment of cells was expected to substantially reduce both intracellular transport and endocytotic mediated uptake of Hst 5. As predicted, uptake of Hst 5 in cells with generalized energy depletion (pre-incubated for 1 h in 10 mM NaN_3_) and in cells with loss of membrane potential (pre-incubated with 500 µM CCCP), had significantly reduced uptake of F-Hst 5 (*P* < 0.0001) when visualized by confocal scanning microscopy (Fig. 7A and C). Cells that did not translocate Hst 5 had abundant cell wall bound peptide (Fig. 7B); thus reduction in uptake was not a result of loss of cell wall binding. However, both azide (only 11.2% of cells contained Hst 5) and CCCP-treated cells (10.9% of cells contained Hst 5) had significantly reduced (*P* < 0.001) cytosolic Hst 5 when compared with control cells (67.3% contained Hst 5) (Fig. 7C), which was consistently found in multiple experiments (*n*= 4). These cells only had cytosolic Hst 5 and were never observed to have vacuolar localized Hst 5, showing that energy-dependent endocytotic uptake did not occur. We next measured Hst 5 uptake parameters in those azide and CCCP-treated cells that did translocate peptide. Energy depleted cells showed high levels of cell wall bound Hst 5 (Fig. 7D), consistent with translocation blockade and accumulation of peptide at the cell wall. Surprisingly, we found that these cells did not undergo the classical sequence of Hst 5 uptake followed by vacuolar expansion and PI entry. Instead, after a cell wall residency time of 800–1000 s, Hst 5 entered the cytosol rapidly and concurrently with vacuolar expansion and PI uptake (Fig. 7D). These cells showed characteristics of direct lytic action or membrane permeabilization in that PI uptake was simultaneous with rapid entry of Hst 5 and accompanying vacuolar expansion. Entry of Hst 5 and PI was uniform throughout the cell, and we did not observe any evidence of single pore formation as previously reported (Mochon and Liu, 2008). Thus, Hst 5 appears to be capable of direct disruption of cellular integrity in about 10% of cells once it has had sufficient residency time within the cell wall.

## Discussion

A major finding in this work is the heterogeneity of Hst 5 intracellular transport mechanisms as well as a range of times required for entry into *C. albicans* cells. Even within a synchronized culture, we observed distinct Hst 5 entry mechanisms (endocytotic and direct cytosolic transport) as well as differing cell wall residency times before entry. In addition, energy-depleted cells had prolonged cell wall retention of Hst 5 followed in some cases by entry through membrane permeabilization. This multiplicity of entry pathways, combined with the dose-dependent rate of Hst 5 uptake, explains some of the reported disparities about the mechanism of Hst 5 candidacidal activity. Due to this heterogeneity in translocation, we found it necessary to examine both single cells as well as cell populations over a time course using defined Hst 5 dosages in order to dissect key temporal events required for its toxicity. Two unifying themes were identified: in all cases Hst 5 cell wall binding precedes (and is required for) uptake; and the majority of Hst 5 translocation is energy-dependent; however, only cytosolic localization results in toxicity. Although we anticipated that autophagy and endocytotic uptake of Hst 5 and subsequent deposition in the vacuole would reduce Hst 5 toxicity, mutants lacking these pathways were fully susceptible to Hst 5 killing. Thus, although Hst 5 can be sequestered in these compartments, it is not an effective means to clear Hst 5 from cells as ongoing cytosolic uptake still occurs.

Fungal vacuoles are organelles functionally similar to mammalian lysosomes in their role in protein degradation and cell detoxification (Ghosh *et al*., 1999). However, they are also ‘crossroads’ organelles (Li and Kane, 2009) in their acute sensitivity to environmental stimuli and their response to stresses including osmotic and ionic shock. If Hst 5 trafficking to the *C. albicans* vacuole functions primarily to sequester and degrade this protein, then we expected that cells carrying mutations that block trafficking to the vacuole would be deficient in Hst 5 detoxification and have higher levels of cell death because of higher cytoplasmic levels of this peptide. However, our data showed that this was not the case. In addition, the enlargement of vacuoles observed in response to Hst 5 may be osmoregulatory and protective in nature. If vacuolar size adaptation is to re-establish intracellular ion homeostatis in response to Hst 5, then cells with mutations resulting in absence of vacuoles also should be hypersensitive to Hst 5. However, these mutants were not more susceptible to Hst 5, illustrating the secondary nature of vacuolar expansion. Our data suggest that vacuolar expansion is a result of movement of water into the vacuole that occurs to balance loss of intracellular ion levels (MacRobbie, 2006) that occurs as a result of cytosolic Hst 5. It has been known for some time that Hst 5 causes rapid efflux of cellular ATP, small molecules and ions (Koshlukova *et al*., 1999); thus vacuolar expansion is expected to be induced by loss of these ions. Our data confirm that vacuolar expansion is secondary to Hst 5-induced ion loss; but does not allow for recovery from toxicity as these cells still undergo cell death (see Fig. 4 and Video S1 and S2). Moreover, we found that PI entry occurs well after initiation of vacuolar expansion and could also be prevented by increasing the concentration of extracellular salt, showing that PI uptake is not through defects/pores in the cell membranes of dead or dying cells (Pina-Vaz *et al*., 2001), but likely passes through ion channels that rely upon ion gradients. Indeed, PI uptake is known to occur through transient openings of channels or point-source burst release of ATP (Arcuino *et al*., 2002). Thus, our data do not support the single breach membrane disruption site proposed by Mochon and Liu (Mochon and Liu, 2008), who suggested internalization into cytoplasm through a unitary disruption on the cell surface. Although this group used supra-physiological concentrations of Hst 5 that may result in anomalous affects, it is more likely that the polar PI staining observed by this group was actually nuclear stain closely approximating the membrane due to shifted organelles following vacuolar expansion.

The use of confocal microscopy to follow trafficking of Hst 5 revealed that many of the cellular effects previously attributed to drug treatment of cells are instead a result of altered cell wall binding and uptake of Hst 5. Thus, the observed reduction of Hst 5 killing in cells treated with energy inhibitors (azide, CCCP) or under low temperatures is not due to decreased cellular ATP *per se*, but instead is a result of reduction of Hst 5 translocation. Once Hst 5 reaches the cytoplasm, loss of cell viability (as measured by PI uptake) is prevented by high extracellular salt, thus showing the crucial feature for toxicity is loss of intracellular ions (cytodialysis). This corresponds with observed vacuolar expansion, Hog1 phosphorylation and glycerol production (Vylkova *et al*., 2007) as cells respond to changes in turgor pressure induced by loss of ions. Whether Hst 5 disrupts the function of specific ion regulatory channels remains to be elucidated.

Interestingly, we found that energy-depleted cells lost most cytosolic (as well as vacuolar) transport of Hst 5, but a small number of cells had simultaneous uptake of Hst 5 and PI indicative of membrane permeabilization or pore formation. This may be a result of high levels of Hst 5 retained at the cell wall leading to non-physiological interactions with the underlying membrane. These data suggest that Hst 5 may have direct membrane effects when applied to cells at sufficiently high concentrations over a longer time span. Thus, the membrane lytic effects of Hst 5 reported previously (Mochon and Liu, 2008) may be due to use of high (> 60 µM) concentrations of this peptide although we found no evidence of a single pore. These findings may have therapeutic implications in that conjugating cell wall binding moieties to Hst 5 in order to enhance cell wall retention may improve its fungicidal activity.

Our finding that cytosolic translocation of Hst 5 is strongly energy-dependent suggests involvement of a membrane-associated transporter dependent upon an energy coupled H^+^ electrochemical gradient. Polyamine permeases in *Saccharomyces cerevisiae* are transmembrane carriers of spermidine, spermine and putrescine (Uemura *et al*., 2007), and also catalyse the uptake of the anticancer drug bleomycin that bears a spermidine substituent at its terminal amine (Aouida *et al*., 2004). Polyamine permeases are energized by the proton motive force generated by yeast Pma1 plasma membrane proton pump and modulated by Trk1p; and their activity also is modified by osmotic stress (Aouida *et al*., 2005). As Hst 5 bears many amine moities, it may utilize *C. albicans* polyamine permeases for its uptake. We are currently investigating this possibility.

It is known that the composition of the microbial cell wall influences the activity of many cationic antimicrobial peptides (Dielbandhoesing *et al*., 1998; Edgerton *et al*., 1998; Peschel *et al*., 1999; Yeaman and Yount, 2003). Recently, it was reported that specific loss of negatively charged N-linked phospho-mannans from the cell wall of *C. albicans* induced resistance to dermaseptin by reducing cell surface peptide binding and entry (Harris *et al*., 2009). In the case of Hst 5, transport is highly dependent on its primary sequence as shown by its translocation deficiency when only two amino acids are substituted while maintaining overall charge (Jang *et al*., 2008). Thus, it is likely that binding interactions between cationic peptides and *C. albicans* cell wall components involve more than net charge, rather Hst 5 (as well as other cationic peptides) may also recognize molecular patterns on the cell surface. *C. albicans* cell wall β-glucans are key components for host binding and signalling through dectin-1 (Gow *et al*., 2007). Also, there is evidence that β-glucans become exposed or ‘unmasked’ at the fungal cell surface during the course of infection (Wheeler *et al*., 2008). We found that Hst 5 binds to β-glucan polysaccharides, but not laminarin biose and triose, suggesting that secondary or ternary conformation of β-glucan containing components of the *Candida* cell wall are involved. Differences in amounts of cell wall β-1,3-glucans among other fungi may modulate the ability of Hst 5 to bind and kill these organisms. The observed reduction in killing of *Candida glabrata* by Hst 5 (Helmerhorst *et al*., 2005) may be a result of lower amounts of cell wall β-1,3-glucan binding units compared with *C. albicans*. Our data also explain the conflicting results on the sensitivity of spheroplasts to Hst 5. Spheroplasts are produced by treating whole cells with a mixture of β-glucanases to digest cell wall glucans and mannans, while cells are placed in sorbitol to ensure osmotic stabilization. Thus, while spheroplasts have reduced β-glucan cell wall binding moities, the presence of sorbitol increases the intracellular uptake of peptide and masks the loss of cell wall Hst 5 binding.

Our finding that Hst 5 utilizes specific cell wall glycans in *C. albicans* suggests that cationic peptides can be designed with motifs for enhanced binding between peptide and cell surface as oral therapeutics for oropharyngeal candidiasis. Our work also highlights the need to identify the fungal transporter or transport mechanism involved in Hst 5 uptake, so that therapeutic peptides could be optimized for *C. albicans* binding and uptake. Such peptides would have increased potency against target fungi, thereby reducing dosage and cost, and rendering them more attractive for clinical use.

## Experimental procedures

### Yeast strains and peptides

All *C. albicans* strains used in this study are listed in Table 1. *C. albicans* CAI4 (Fonzi and Irwin, 1993) was used for all experiments as a control strain; *myo5*Δ/Δ strain was generously provided by Dr Malcolm Whiteway, McGill University, Montreal, Canada; *vps21*Δ/Δ, *ypt72*Δ/Δ, and *atg*Δ/Δ strains were kindly provided by Dr Glen E. Palmer, Louisiana State University, New Orleans, USA. Yeast were cultured overnight at 30°C in 10 ml of yeast extract-peptone-dextrose (YPD) broth (Qbiogene), then diluted in 10 ml of YPD broth to an OD_600_ = 0.2, and regrown for an additional 4 h with shaking at 30°C to reach an OD_600_ = 1 to obtain a mid-log phase cultures. Hst 5, biotin-labelled Hst 5 (BHst 5) and FITC-labelled Hst 5 (F-Hst 5) were synthesized by Genemed Synthesis. (San Francisco, CA). BHst 5 and F-Hst 5 were verified to have similar biological activity as unlabelled Hst 5 by candidacidal assays (Helmerhorst *et al*., 1999; Koshlukova *et al*., 2000).

### Hst 5 binding assays with *C. albicans* cells

Binding of Hst 5 to the surface of *C. albicans* cells was examined by assessing the effect of fungal cell wall components on killing and cell wall binding activity of Hst 5. BHst 5 (20 µl) or Hst 5 (20 µl) (final concentration = 31 µM) was added to 80 µl of each polysaccharide [1.125–20 mg ml^−1^ in 10 mM sodium phosphate buffer, pH 7.4 (NaPB)] including laminarin (β-1,3-glucan polymer; Sigma), sialic acid (Sigma), pustulan (β-1,6-glucan polymer; Calbiochem) or mannan (mannose polymer; Sigma) and incubated with *C. albicans* cells for 1 h at 30°C. The effect of polysaccharides on killing and cell wall binding of Hst 5 was assessed by microdilution plate candidacidal assay and cell wall binding assay described below.

### Candidacidal assays of Hst 5

Candidacidal activities of Hst 5 against *C. albicans* cells were tested using microdilution plate candidacidal assays as we have previously described (Baev *et al*., 2003). Briefly, *Candida* cells (1 × 10^6^ cells ml^−1^) were mixed with Hst 5 at 30°C for 1 h, diluted in 10 mM NaPB, and aliquots of 500 cells were spread onto YNB (yeast nitrogen base) agar plates and incubated for 48 h at room temperature. In some experiments, cells were grown in YPD broth containing 1 or 2 ng ml^−1^ of caspofungin (Merck) overnight. Cell survival was expressed as a percentage compared with untreated control cells, and the loss of viability was calculated as [1 − (number of colonies from peptide-treated cells/number of colonies from control cells)] × 100. Assays were performed in triplicate for each strain. For the effects of endocytosis inhibitory drugs on killing of Hst 5, cells (1 × 10^6^ cells ml^−1^) were pre-incubated with 10 µM of jasplakinolide, latrunculin A or cytochalasin A (Sigma) for 1 h at 37°C.

### Cell wall binding and translocation assays of Hst 5

Localization of Hst 5 in the *C. albicans* cell wall or cytosol was examined by two sequential cellular fractionation steps consisting of β-mercaptoethanol (β-ME) cell wall extraction, followed by cytosolic fractionation, as described previously (Jang *et al*., 2008). Briefly, early-log-phase cells (1 × 10^7^) were washed twice with 10 mM NaPB and suspended in 1 ml of 10 mM NaPB, and BHst 5 were added to a final concentration of 31 µM. In some experiments, cells were pre-incubated with NaN_3_ (Sigma) or cyanide *m*-chlorophenylhydrazone (CCCP) (Sigma) for 1 h at 30°C. After incubation for 30 min, cell wall components were extracted by incubation of the cell suspension in ammonium carbonate buffer (pH 8.0) containing 1% (vol/vol) β-ME for 30 min at 37°C. Cell wall extracts were collected following centrifugation at 3500 r.p.m., washed with 10 mM NaPB, and the cell pellet was resuspended in 300 µl of cold lysis buffer supplemented with protease inhibitors (10 mM NaPB, 1 mM phenylmethylsulfonyl fluoride, 1 mM EDTA, 1 µg ml^−1^ aprotinin, 1 µg ml^−1^ pepstatin A, 1 µg ml^−1^ leupeptin and 1 µg ml^−1^ benzamidine). Cell lysates were prepared using a Fastprep apparatus at 4°C. The cytosolic fraction was collected following centrifugation at 13 000 r.p.m. for 10 min. Protein concentration for each cell wall and cytosolic protein extract was measured by BCA assay (Pierce) and cell wall or cytosolic proteins (10 µg) were separated using 16% Tricine SDS-PAGE, immunoblotted with Streptavidin-HRP (Pierce), and developed by ECL. Quantification of BHst 5 in cell wall and cytosolic extracts was determined by analysis using Quantity One software (version 4.2).

### Affinity chromatography analysis of Hst 5 binding to polysaccharides

Binding of Hst 5 to selected polysaccharides found in the yeast cell wall was tested using epoxy-activated Sepharose 6B (Sigma) beads coupled to each polysaccharide (Lu *et al*., 1995). Beads (1 g) pre-equilibrated in coupling buffer (10 mM bicarbonate, pH 9.5) were reacted with laminarin, sialic acid, pustulan or mannan (20 mg) at room temperature for 24 h. Any remaining reactive groups were blocked by incubating beads in 1 M ethanolamine in coupling buffer for 4 h. Polysaccharide-coupled beads (1 ml) were placed in a column (5 × 1 cm) and equilibrated with 10 mM NaPB before loading BHst 5 (100 µg). Excess unbound Hst 5 was removed from the column by extensive washing using 10 mM NaPB. Bound BHst 5 was eluted from each column with 0.010–1 M NaCl, and concentration of eluted protein was determined by BCA assay (Pierce) to calculate the percent bound Hst 5 to loaded protein. Eluted fractions were loaded onto a Minifold II Slot Blot System (Whatman), and visualized by streptavidin-HRP (Pierce) and ECL.

### Binding of Hst 5 with purified cell wall and laminarinase digestion of cell walls of *C. albicans*

Purification of cell walls of *C. albicans* were performed as described (Maddi *et al*., 2009). Briefly, cells were cultured in 500 ml of YPD and collected in a mid-log phase cultures by centrifugation at 4000 *g* for 5 min. The collected cells were then washed once in ice-cold PBS and resuspended in 10 ml of ice-cold PBS (approximate titre of 1.0 × 10^9^ cells ml^−1^) and subjected to 30 cycles at 6.0 speed for 20 s in a FastPrep machine maintained at 4°C, with 1 min cooling on ice after each cycle. The resulting lysate was collected and centrifuged at 4000 *g* for 5 min at 4°C. The pellets were then resuspended in 1% SDS in PBS and boiled for 15 min. The samples were cooled to room temperature and centrifuged at 10 000 *g* for 5 min. The pellets were washed twice with ice-cold PBS and resuspended in ammonium carbonate buffer (pH 8.0) containing 1% (vol/vol) β-ME and incubated at 37°C with shaking at 150 r.p.m. for 30 min. The extract was centrifuged at 10 000 *g* for 5 min at 4°C and the pellet was collected as ‘purified cell wall (PCW)’. The pellet was washed twice with ice-cold PBS and once with ice-cold distilled water at 10 000 *g* for 5 min. The PCW preparations were lyophilized. To determine inhibitory effect of released polysaccharides from PCW on binding activity of Hst 5 to *Candida* cell wall, PCW was incubated in 100 mM sodium acetate buffer (pH 5.5) with 0.05 U laminarinase (Sigma) for 1 h. After centrifugation at 10 000 *g*, supernatant containing released polysaccharides consisting of β-glucans were collected and boiled for 10 min at 100°C to inhibit enzyme activity. Amount of laminarinase digest was calculated by measuring loss of PCW (pellets). The purity of polysaccharide in the laminarinase digest was determined by steric exclusion chromatography at the Complex Carbohydrate Research Center at the University of Georgia and found to consist of one major peak of 7 kDa (90%) and three minor peaks of smaller molecular weights that could be dimers and trimers. Hst 5 (20 µl) (final concentration = 31 µM) was added to 80 µl of released polysaccharides (2–8 mg ml^−1^) and incubated with *C. albicans* cells for 30 min at 30°C. Cells were washed with 10 mM NaPB twice, then cell wall bound Hst 5 was eluted with 100 mM NaCl and subjected to 16% Tricine SDS-PAGE. To test binding activity of Hst 5 to PCW or laminarinase digestion of PCW, Hst 5 (final concentration = 31.5 µM) was incubated with PCW (20 mg ml^−1^) or laminarinase digestion of PCW (20 mg ml^−1^) for 10 min. Unbound Hst 5 in supernatant was collected and bound Hst 5 were eluted from the pellets by addition of 10 mM NaPB with 100 mM NaCl and then subjected to 16% Tricine SDS-PAGE.

### Live cell imaging of Hst 5 trafficking by confocal microscopy

All data were acquired using living cell images of *C. albicans* cells attached to concanavalin A (Sigma, 1 mg ml^−1^ solution in water) coated coverglass in chambered wells (Lab-Tek II). Cells (1 × 10^6^) were deposited in each well, and 1 ml of 10 mM NaPB containing 5 µg PI (Sigma) and F-Hst 5 (final concentration 15.5 µM and 31 µM) was added to the chamber. For energy depletion experiments, cells were pre-incubated with 10 mM NaPB containing 10 mM NaN_3_ or 500 µM CCCP at 30°C for 60 min then washed for addition of F-Hst 5. For cells pretreated with F-Hst 5, 31 µM F-Hst 5 was added first for 5 min and then 10 mM NaN_3_, 100 mM NaCl or 1 M sorbitol was added to the well. Confocal images were acquired with a Zeiss LSM510 Meta Confocal Microscope (Carl Zeiss, Germany) using Plan Apochromat 63X/1.4 objectives. For simultaneous detection of F-Hst 5 and PI, the 488 nm line of the argon ion laser and a 561 nm DPSS laser were directed over an HFT UV/488/561 beam splitter, and fluorescence was detected using Mirror or NFT 565 beam splitter in combination with a BP 500-550 band pass filter for F-Hst 5 and an LP 575 or BP 650-710 band pass filter for PI detection. ImageJ software was used for image acquisition, analysis, and kymograph construction.

## Supporting Information

**Video S1**

Intracellular trafficking of F-Hst 5 (15.5 μM) and PI uptake in *C. albicans*. After addition of F-Hst 5 to a *Candida* cell suspension containing PI (5 μg/ml), images were recorded by time-lapse confocal microscopy with frames recorded every 10 seconds for a total of 30 min and converted to MOV file (ImageJ software) using frames with 20 second intervals. Heterogeneity of Hst 5 trafficking to the cytosol or vacuole is observed, however cytosolic localization precedes vacuolar expansion (shown in selected cells with arrows) and propidium Iodide entry.

**Video S2**

Intracellular trafficking of F-Hst 5 (31 μM) and PI uptake in *C. albicans*. After addition of F-Hst 5 to a *Candida* cell suspension containing PI (5 μg/ml), images were recorded by time-lapse confocal microscopy with frames recorded every 10 seconds for a total of 30 min and converted to MOV file (ImageJ software) using frames with 20 second intervals. Hst 5 trafficking to the cytosol or vacuole precedes vacuolar expansion (shown in selected cells with arrows) and propidium Iodide uptake.

## References

[b1] Aouida M, Page N, Leduc A, Peter M, Ramotar D (2004). A genome-wide screen in *Saccharomyces cerevisiae* reveals altered transport as a mechanism of resistance to the anticancer drug bleomycin. Cancer Res.

[b2] Aouida M, Leduc A, Poulin R, Ramotar D (2005). AGP2 encodes the major permease for high affinity polyamine import in *Saccharomyces cerevisiae*. J Biol Chem.

[b3] Arcuino G, Lin JH, Takano T, Liu C, Jiang L, Gao Q (2002). Intercellular calcium signaling mediated by point-source burst release of ATP. Proc Natl Acad Sci USA.

[b4] Baev D, Li XS, Dong J, Keng P, Edgerton M (2002). Human salivary histatin 5 causes disordered volume regulation and cell cycle arrest in *Candida albicans*. Infect Immun.

[b5] Baev D, Rivetta A, Li XS, Vylkova S, Bashi E, Slayman CL, Edgerton M (2003). Killing of *Candida albicans* by human salivary histatin 5 is modulated, but not determined, by the potassium channel TOK1. Infect Immun.

[b6] Baev D, Rivetta A, Vylkova S, Sun JN, Zeng GF, Slayman CL, Edgerton M (2004). The TRK1 potassium transporter is the critical effector for killing of *Candida albicans* by the cationic protein, Histatin 5. J Biol Chem.

[b7] Bensen ES, Filler SG, Berman J (2002). A forkhead transcription factor is important for true hyphal as well as yeast morphogenesis in *Candida albicans*. Eukaryot Cell.

[b8] Castagnola M, Inzitari R, Rossetti DV, Olmi C, Cabras T, Piras V (2004). A cascade of 24 histatins (histatin 3 fragments) in human saliva. Suggestions for a pre-secretory sequential cleavage pathway. J Biol Chem.

[b9] Chaffin WL, Lopez-Ribot JL, Casanova M, Gozalbo D, Martinez JP (1998). Cell wall and secreted proteins of *Candida albicans*: identification, function, and expression. Microbiol Mol Biol Rev.

[b10] Den Hertog AL, Wong Fong Sang HW, Kraayenhof R, Bolscher JG, Van't Hof W, Veerman EC, Nieuw Amerongen AV (2004). Interactions of histatin 5 and histatin 5-derived peptides with liposome membranes: surface effects, translocation and permeabilization. Biochem J.

[b11] Dielbandhoesing SK, Zhang H, Caro LH, van der Vaart JM, Klis FM, Verrips CT, Brul S (1998). Specific cell wall proteins confer resistance to nisin upon yeast cells. Appl Environ Microbiol.

[b12] Edgerton M, Koshlukova SE, Lo TE, Chrzan BG, Straubinger RM, Raj PA (1998). Candidacidal activity of salivary histatins. Identification of a histatin 5-binding protein on *Candida albicans*. J Biol Chem.

[b13] Efe JA, Botelho RJ, Emr SD (2005). The Fab1 phosphatidylinositol kinase pathway in the regulation of vacuole morphology. Curr Opin Cell Biol.

[b14] Fonzi WA, Irwin MY (1993). Isogenic strain construction and gene mapping in *Candida albicans*. Genetics.

[b15] Ghosh M, Shen J, Rosen BP (1999). Pathways of As(III) detoxification in *Saccharomyces cerevisiae*. Proc Natl Acad Sci USA.

[b16] Gow NA, Netea MG, Munro CA, Ferwerda G, Bates S, Mora-Montes HM (2007). Immune recognition of *Candida albicans* beta-glucan by dectin-1. J Infect Dis.

[b17] Gyurko C, Lendenmann U, Troxler RF, Oppenheim FG (2000). *Candida albicans* mutants deficient in respiration are resistant to the small cationic salivary antimicrobial peptide histatin 5. Antimicrob Agents Chemother.

[b18] Harris M, Mora-Montes HM, Gow NA, Coote PJ (2009). Loss of mannosylphosphate from *Candida albicans* cell wall proteins results in enhanced resistance to the inhibitory effect of a cationic antimicrobial peptide via reduced peptide binding to the cell surface. Microbiology.

[b19] Helmerhorst EJ, Breeuwer P, van't Hof W, Walgreen-Weterings E, Oomen LC, Veerman EC (1999). The cellular target of histatin 5 on *Candida albicans* is the energized mitochondrion. J Biol Chem.

[b20] Helmerhorst EJ, Troxler RF, Oppenheim FG (2001). The human salivary peptide histatin 5 exerts its antifungal activity through the formation of reactive oxygen species. Proc Natl Acad Sci USA.

[b21] Helmerhorst EJ, Venuleo C, Beri A, Oppenheim FG (2005). *Candida glabrata* is unusual with respect to its resistance to cationic antifungal proteins. Yeast.

[b22] Hoffmann JA, Kafatos FC, Janeway CA, Ezekowitz RA (1999). Phylogenetic perspectives in innate immunity. Science.

[b23] Hohmann S (2002). Osmotic stress signaling and osmoadaptation in yeasts. Microbiol Mol Biol Rev.

[b24] Jang WS, Kim HK, Lee KY, Kim SA, Han YS, Lee IH (2006). Antifungal activity of synthetic peptide derived from halocidin, antimicrobial peptide from the tunicate, *Halocynthia aurantium*. FEBS Lett.

[b25] Jang WS, Li XS, Sun JN, Edgerton M (2008). The P-113 fragment of histatin 5 requires a specific peptide sequence for intracellular translocation in *Candida albicans*, which is independent of cell wall binding. Antimicrob Agents Chemother.

[b26] Johnston DA, Eberle KE, Sturtevant JE, Palmer GE (2009). Role for endosomal and vacuolar GTPases in *Candida albicans* pathogenesis. Infect Immun.

[b27] Kapteyn JC, Hoyer LL, Hecht JE, Muller WH, Andel A, Verkleij AJ (2000). The cell wall architecture of *Candida albicans* wild-type cells and cell wall-defective mutants. Mol Microbiol.

[b28] Koshlukova SE, Lloyd TL, Araujo MW, Edgerton M (1999). Salivary histatin 5 induces non-lytic release of ATP from *Candida albicans* leading to cell death. J Biol Chem.

[b29] Koshlukova SE, Araujo MW, Baev D, Edgerton M (2000). Released ATP is an extracellular cytotoxic mediator in salivary histatin 5-induced killing of *Candida albicans*. Infect Immun.

[b30] Li SC, Kane PM (2009). The yeast lysosome-like vacuole: endpoint and crossroads. Biochim Biophys Acta.

[b31] Li XS, Sun JN, Okamoto-Shibayama K, Edgerton M (2006). *Candida albicans* cell wall ssa proteins bind and facilitate import of salivary histatin 5 required for toxicity. J Biol Chem.

[b32] Lu CF, Montijn RC, Brown JL, Klis F, Kurjan J, Bussey H, Lipke PN (1995). Glycosyl phosphatidylinositol-dependent cross-linking of alpha-agglutinin and beta 1,6-glucan in the *Saccharomyces cerevisiae* cell wall. J Cell Biol.

[b33] MacRobbie EA (2006). Osmotic effects on vacuolar ion release in guard cells. Proc Natl Acad Sci USA.

[b34] Maddi A, Bowman SM, Free SJ (2009). Trifluoromethanesulfonic acid-based proteomic analysis of cell wall and secreted proteins of the ascomycetous fungi *Neurospora crassa* and *Candida albicans*. Fungal Genet Biol.

[b35] Martinez JP, Gil ML, Lopez-Ribot JL, Chaffin WL (1998). Serologic response to cell wall mannoproteins and proteins of *Candida albicans*. Clin Microbiol Rev.

[b36] Mochon AB, Liu H (2008). The antimicrobial peptide histatin-5 causes a spatially restricted disruption on the *Candida albicans* surface, allowing rapid entry of the peptide into the cytoplasm. PLoS Pathog.

[b37] Oberholzer U, Marcil A, Leberer E, Thomas DY, Whiteway M (2002). Myosin I is required for hypha formation in *Candida albicans*. Eukaryot Cell.

[b38] Oberholzer U, Nantel A, Berman J, Whiteway M (2006). Transcript profiles of *Candida albicans* cortical actin patch mutants reflect their cellular defects: contribution of the Hog1p and Mkc1p signaling pathways. Eukaryot Cell.

[b39] Oppenheim FG, Xu T, McMillian FM, Levitz SM, Diamond RD, Offner GD, Troxler RF (1988). Histatins, a novel family of histidine-rich proteins in human parotid secretion. Isolation, characterization, primary structure, and fungistatic effects on *Candida albicans*. J Biol Chem.

[b40] Palmer GE, Kelly MN, Sturtevant JE (2007). Autophagy in the pathogen *Candida albicans*. Microbiology.

[b41] Peschel A, Otto M, Jack RW, Kalbacher H, Jung G, Gotz F (1999). Inactivation of the dlt operon in *Staphylococcus aureus* confers sensitivity to defensins, protegrins, and other antimicrobial peptides. J Biol Chem.

[b42] Pina-Vaz C, Sansonetty F, Rodrigues AG, Costa-Oliveira S, Tavares C, Martinez-de-Oliveira J (2001). Cytometric approach for a rapid evaluation of susceptibility of *Candida* strains to antifungals. Clin Microbiol Infect.

[b43] Raj PA, Edgerton M, Levine MJ (1990). Salivary histatin 5: dependence of sequence, chain length, and helical conformation for candidacidal activity. J Biol Chem.

[b44] Stevens HC, Nichols JW (2007). The proton electrochemical gradient across the plasma membrane of yeast is necessary for phospholipid flip. J Biol Chem.

[b45] Sun JN, Li W, Jang WS, Nayyar N, Sutton MD, Edgerton M (2008). Uptake of the antifungal cationic peptide Histatin 5 by *Candida albicans* Ssa2p requires binding to non-conventional sites within the ATPase domain. Mol Microbiol.

[b46] Uemura T, Kashiwagi K, Igarashi K (2007). Polyamine uptake by DUR3 and SAM3 in *Saccharomyces cerevisiae*. J Biol Chem.

[b47] Veerman EC, Nazmi K, Van't Hof W, Bolscher JG, Den Hertog AL, Nieuw Amerongen AV (2004). Reactive oxygen species play no role in the candidacidal activity of the salivary antimicrobial peptide histatin 5. Biochem J.

[b48] Veerman EC, Valentijn-Benz M, Nazmi K, Ruissen AL, Walgreen-Weterings E, van Marle J (2007). Energy depletion protects *Candida albicans* against antimicrobial peptides by rigidifying its cell membrane. J Biol Chem.

[b49] Vylkova S, Jang WS, Li W, Nayyar N, Edgerton M (2007). Histatin 5 initiates osmotic stress response in *Candida albicans* via activation of the Hog1 mitogen-activated protein kinase pathway. Eukaryot Cell.

[b50] Weisman LS (2003). Yeast vacuole inheritance and dynamics. Annu Rev Genet.

[b51] Wheeler RT, Kombe D, Agarwala SD, Fink GR (2008). Dynamic, morphotype-specific *Candida albicans* beta-glucan exposure during infection and drug treatment. PLoS Pathog.

[b52] Xu Y, Ambudkar I, Yamagishi H, Swaim W, Walsh TJ, O'Connell BC (1999). Histatin 3-mediated killing of *Candida albicans*: effect of extracellular salt concentration on binding and internalization. Antimicrob Agents Chemother.

[b53] Yeaman MR, Yount NY (2003). Mechanisms of antimicrobial peptide action and resistance. Pharmacol Rev.

